# Supplementary material for: Clinical Prediction Models for Recurrence in Patients with Resectable Grade 1 and 2 Sporadic Non-Functional Pancreatic Neuroendocrine Tumors: A Systematic Review
Source: Cancers (Basel). 2023 Feb 28;15(5):1525. doi: 10.3390/cancers15051525 (PMC10001130; doi:10.3390/cancers15051525)
Supplement: Supplementary file 1 [file cancers-15-01525-s001.zip › cancers-2175456-supplementary.pdf]

Table S1. Critical appraisal table

		Ballian (2009)	Dong (2021)	Fisher (2019)	Genç (2018)	Heidsma (2021)	Liu (2013)	Primavesi (2020)	Pulvirenti (2021)	Sho (2018)	Sun (2019)	Viúdez (2016)	Wei (2021)	Zhou (2017)	Zou (2020)
**Source of data and participant selection**	**Applicability**	✓	✓	✓	✓	✓	✓	✓	✓	✓	✓	✓	✓	✓	✓
	**Risk of Bias**	✓	✓	✓	✓	✓	✓	✓	✓	✓	✓	✓	✓	✓	✓
**Predictor selection**	**Applicability**	✓	✓	✓	✓	✓	✓	✓	✓	✓	✓	X	X	✓	✓
	**Risk of Bias**	✓	✓	✓	✓	✓	✓	✓	✓	✓	✓	✓	✓	✓	✓
**Appropriate outcome determination**	**Applicability**	✓	✓	✓	✓	✓	✓	✓	✓	✓	✓	✓	✓	✓	✓
	**Risk of Bias**	✓	✓	X	✓	✓	✓	✓	✓	✓	✓	✓	✓	✓	✓
**Analysis: development, performance and evaluation**	**Risk of Bias**	X	X	X	X	✓	X	X	X	X	X	X	X	X	X
**Overall judgment Risk of Bias**		X	X	X	X	✓	X	X	X	X	X	X	X	X	X

✓ = low risk of bias; X = high risk of bias
